# An all-endo Approach to Complete Ureteral Duplications Complicated by Ureterocele and/or Vesicoureteral Reflux: Feasibility, Limitations, and Results

**DOI:** 10.1155/2011/103067

**Published:** 2011-12-14

**Authors:** A. Calisti, M. L. Perrotta, R. Coletta, C. Olivieri, V. Briganti, L. Oriolo, R. Fabbri

**Affiliations:** ^1^Pediatric Surgery and Urology Unit, San Camillo Hospital, Circ. Gianicolense 87, 00152 Rome, Italy; ^2^Radiology Department, San Camillo Hospital, Circ. Gianicolense 87, 00152 Rome, Italy

## Abstract

*Purpose*. Totally endoscopic management (all-endo) of patients with a duplicated renal system (DS) associated with severe vesicoureteral reflux (VUR) or obstructive ureterocele (UC) is an attractive alternative to traditional open procedures. The authors discuss feasibility and results of an all-endo approach on a consecutive series of patients. *Methods*. From 1999 to 2009, all patients with a complete DS associated with UC and/or VUR were proposed for primary all-endo approach. UC puncture was performed using a 3 Fr Bugbee electrode. Deflux (dextranomer/hyaluronic acid copolymer) injection was administered for VUR. The need for secondary surgery was evaluated on followup. *Results*. Of the 62 patients recruited, 46 were treated using a primary all-endo approach and 16 patients received no treatment. Of the 46 treated patients with 56 affected renal units, 32 (97%) UCs collapsed following puncture and 29 (63%) VURs were resolved or downgraded. Secondary VUR occurred in 13 (39%) renal units. Secondary surgery was performed on 23 (41%) renal units. *Conclusion*. The all-endo approach for VUR in DS is an effective therapeutic option. UC collapse was achieved by puncture in most of the patients; secondary VUR was the main complication in a small group of extravesical UC.

## 1. Introduction

There is wide debate on the management of patients with a complete duplicated pyeloureteral system (DS) associated with ureterocele (UC) and/or major vesicoureteral reflux (VUR), and consensus on this matter has not yet been reached [1–5]. This may be due to the wide spectrum of anatomical and clinical features observed in affected patients and the need for an individualized approach. As far as the endoscopic approach to treatment is concerned, the use of UC puncture to relieve obstruction, control infection, and recover renal function is hampered by controversial outcomes and side effects [6]. Secondary VUR and the need for subsequent intravesical surgery have been reported by several studies [6, 7]. In contrast, a conservative approach to the treatment of certain types of UC has been gaining favour [3]. Treatment of VUR associated with a duplex pyeloureteral system, with or without concomitant UC, is another matter for debate. Endoscopic intrameatal injection of bulking agents was reported to be less effective than in a single system when used to treat VUR [8]. During the last decade, the treatment policy at our institution has been to consider minimally invasive all-endo management as the primary option for this group of urinary tract anomalies. The aim of our study was to investigate the feasibility, limitations, and possible advantages of this approach.

## 2. Methods

### 2.1. Patients and Treatments

From 1999 to 2009, patients with unilateral or bilateral complete DS associated with UC and/or VUR were recruited. Informed consent was acquired for each patient. Preliminary examinations included ultrasonography and voiding cystourethrogram (VCUG). The grade and side of VUR were recorded. Dilated duplicated system, position (intra- or extravesical), and size of UC were established. A 99mTc-mercaptoacetyltriglycine (MAG3) diuretic renal scan was used in all UCs with a dilated upper system. A 99mTc-dimercaptosuccinic acid (DMSA) renal scan was performed in all VUR to evaluate scar formation. Urinary magnetic resonance imaging was undertaken in patients with unclear anatomy and poor renal function. After initial conservative management, a primary all-endo treatment approach was elected for selected patients. Common indications to perform UC puncture were a dilated upper system and recurrent urinary infections or an obstructive renographic pattern in a still functional renal moiety. Endoscopic treatment was indicated for patients with persistent grade ≥III VUR and recurrent urinary tract infections following antibiotic prophylaxis. Three treatment options were available: puncture alone, puncture and Deflux (Oceana Therapeutics, Inc., Edison, USA. [submucosal, intrameatal dextranomer/hyaluronic acid copolymer]) injection, and Deflux injection only. UC puncture was performed on the lower portion using a 3 Fr Bugbee electrode. Deflux injection in the refluxing ureter was the preferred endoscopic procedure.

UC size and upper tract dilatation were monitored twice a month after endoscopic puncture using ultrasonography; possible occurrence of secondary VUR was monitored by VCUG at one month. The MAG3 diuretic scan was repeated after 3 months. Results of VUR treatment were monitored by VCUG 3–6 months after Deflux injection. Patients underwent a repeat DMSA scan 6 months after conservative treatment and 12 months after successful endoscopic management. Secondary open surgery was considered if the endoscopic approach was unsuccessful. Whenever a simultaneous all-endo treatment of UC and VUR was performed, a control MAG3 scan was only used to check postoperative split function.

### 2.2. Statistical Analysis

The outcome of a primary all-endo approach was correlated with DS variants (the grade of associated VUR; position and anatomy of associated UC). Data were analyzed with GraphPad InStat software (version 3.10) (GraphPad Software, Inc., La Jolla, USA). The chi-square test used for univariate comparisons. Fisher's exact test was used comparisons of categorical variables.

Multiple regression tests were used for factors influencing outcome. *P* < 0.05 was considered significant.

## 3. Results

### 3.1. Patient Characteristics

A total of 62 patients, with male : female ratio of 43 : 19 and a mean age at referral of 18 months, (range 1–96 months), were recruited. Detection of hydronephrosis by antenatal ultrasonography led to the enrollment of 45 patients (73%); all other patients (*n* = 17, 27%) were enrolled because of a history of febrile urinary tract infections. Among the 62 patients with documented DS, 40 patients had a UC of the upper moiety. Twenty-three UCs were on the right side (extravesical *n* = 12; intravesical *n* = 11), and 17 were on the left side (extravesical *n* = 9; intravesical *n* = 8). No bilateral cases were observed. VUR was associated with DS in 44 patients, occurring on the right side in 26 patients (grade II *n* = 3; grade III *n* = 10; grade IV *n* = 9; grade V *n* = 4) and on the left side in 18 patients (grade II *n* = 6, grade III *n* = 3, grade IV *n* = 8, and grade V *n* = 2). In one patient, VUR affected both renal districts of the left DS. No treatment was required in 16 patients. Among these patients there were six nonobstructive small intravesical UCs and one extravesical UC corresponding to a multicystic upper moiety. Asymptomatic grade I–III VUR affecting 12 lower renal moieties reduced spontaneously within 6 months after diagnosis.

### 3.2. Treatments

An all-endo primary treatment approach was elected for 46 patients (32 boys and 14 girls). Of these patients, 30 (65%) were enrolled following antenatal ultrasonography diagnosis of DS and/or UC at birth. Mean age at first treatment was 39 months (range 1–95 months). Among these patients, 9 had complete bilateral DS and 37 had complete unilateral DS. Forty-three unilateral UCs were observed. Twenty extravesical UCs were punctured; 12 of these UCs were associated with grade ≥III VUR in the lower pole and these patients underwent simultaneous endoscopic VUR correction. Thirteen intravesical UCs were punctured, 11 of which were associated with grade ≥III VURs in the lower moiety, which were treated endoscopically during the procedure. VUR not associated with UC was recorded in 23 renal moieties in 22 patients with DS. The lower renal moiety was involved in 21 of these patients, and both renal moieties were involved in the remaining patient. Deflux injections were administered to each affected renal unit. The procedure was repeated 4 months after the initial injection in 6 patients for a persisting grade ≥III VUR or for a lower grade associated with UTI. The outcome of the all-endo primary treatment approach to 56 renal units affected by UC and/or grade ≥III VUR is shown in Table 1.

In the 46 patients with DS associated with UC and/or VUR, the primary all-endo treatment was successful and resolutive in 23 (50%). For a further 17 (37%) patients this treatment strategy resolved breakthrough urinary tract infection and urinary obstruction and delayed the need for open reconstructive surgery. Only 6 patients (13%) with persistent severe VUR required secondary total or partial demolitive surgery for severe renal dysplastic changes. Factors affecting the outcome of the all-endo management approach and the rate of secondary surgery are shown in Table 2.

Endoscopic treatment of VUR by Deflux injection and puncture or Deflux injection alone was successful in 63% of renal units treated (*n* = 29). The outcome of all-endo management was significantly influenced by the grade of VUR (*P* = 0.0261). UC collapse after puncture occurred in 32/33 renal units (97%), but secondary VUR occurred in only 13 of these patients (39%). The occurrence of this complication was significantly correlated with UC position and anatomy (extravesical more than intravesical; *P* = 0.0315). UC position and anatomy also significantly influenced the need for secondary open surgical procedures in 23/56 renal units (41%; *P* = 0.019). In 14 of them there was an extravesical UC, in 12 an initial high-grade (≥IV) VUR in the lower pole. Twenty-five secondary surgical procedures were required (Table 3) mainly due to persisting high-grade primary VUR in the lower pole. Surgery was rarely required to treat secondary iatrogenic VUR in the upper pole occurring after UC puncture.

## 4. Discussion

Recent thinking on an endoscopic approach to DS associated with UC and/or VUR continues to be controversial. Surgery at the bladder level has been advocated for large extravesical UC of the upper pole associated with high-grade VUR in the lower pole [9–11]. After introduction of laparoscopic approach, upper pole nephrectomy has been increasingly advised for isolated extravesical UC with absent or poor renal function in the corresponding renal moiety without VUR in the lower pole. This aggressive attitude must be tempered considering that many unobstructed, uninfected UC with conserved renal function or a nonfunctioning dysplastic upper pole may be candidates for nonsurgical therapy, [3] with many cases reported to resolve spontaneously [12]. The endoscopic puncture of an obstructive UC leads to recovery of renal function in a proportion of patients [7, 13, 14]. Additionally, VUR in the lower moiety associated with upper pole UC has been reported to resolve or downgrade following puncture in 48% of patients [5]. Criticism of primary UC puncture is mainly based on the high rate of reoperation, which is required in approximately half of all cases of extravesical UCs and whenever lower pole VUR is associated with UC. Use of this procedure is therefore restricted to the emergency treatment of the UC or the elective management of some patients with intravesical UCs. A recent meta-analysis of surgical practice patterns in the endoscopic management of UC concluded that the UC location (extravesical versus intravesical) and renal anatomy, together with preoperative lower pole VUR, are proxies for trigonal distortion, which is accompanied by an increased risk of reoperation [1]. As far as VUR in DS is concerned, the outcome in patients with lower grade VUR was similar to that seen in patients with a single renal system, and this justifies the conservative management approach [2]. Indeed, endoscopic treatment of higher-grade lower pole VUR in patients with DS has been reported to have a success rate of 73% [4, 15–17]. Whenever UC of the upper pole is associated with lower moiety VUR, over 70% of patients respond to endoscopic correction after UC endoscopic puncture [5]. In conclusion a conservative approach can be the primary option for small nonobstructive intravesical UC and for those extravesical UC corresponding to a multicystic upper moiety without any bladder outlet obstruction. Even asymptomatic mild VUR in the lower moiety, can be preferably left alone, and spontaneous resolution can be expected. In all other cases of complete ureteral duplications complicated by UC and/or VUR an all-endo approach is a feasible option and reduces the need for major open or laparoscopic surgery. Difficulties correlated with individual anatomical variations and sometimes with severe trigonal distortion must be always considered; they require experienced hands and may demand multiple procedures.

## Figures and Tables

**Table 1:** Details of the *all-endo* treatment approach for duplicated renal unit.

Pathology	Number of renal units	Procedure	Number of renal units interested (%)			
			UC collapses after puncture	VUR resolved or downgraded	Secondary VUR after UC puncture	Secondary surgery
DS-UC	10	Puncture	9 (90)	—	5 (50)	5 (50)
DS-UC-VUR	23	Puncture & Deflux injection	23 (100)	11 (48)	8 (35)	13 (57)
DS-VUR	23	Deflux injection	—	18 (78)	—	5 (22)
Total	56	—	32/33 (97)	29/46 (63)**^a^**	13/33 (39)	23/56 (41)

**^a^**2 Deflux injections were performed in 6 patients;* DS*: duplicated renal system;* UC*: ureterocele;* VUR*: vesicoureteral reflux.

**Table 2:** Factors affecting the outcome of the *all-endo* approach and the need for secondary surgical intervention.

Outcome	No. of renal units (%)	Ureterocele position and anatomy	VUR grade in lower moiety
VUR resolved or downgraded	29/46 (63)	NS	*P* = 0.0261
Secondary VUR after UC puncture	13/33 (39)	*P* = 0.0315	NS
Required open surgery	23/56 (41)	*P* = 0.019	NS

*NS*: not statistically significant;* UC*: ureterocele*; VUR*: vesicoureteral reflux.

**Table 3:** Type and details of secondary surgery performed.

Type of surgical intervention	No. of renal units	Indications
Double vesicoureteral reimplant	18	Persistent lower pole VUR (*n* = 14) and/or secondary upper pole VUR (*n* = 6)
Upper pole nephrectomy	1	Secondary upper pole VUR and severe dysplasia
Upper pole nephrectomy and lower pole reimplant	2	Upper pole dysplasia and persistent lower pole VUR (*n* = 2)
Pyelopyelic anastomosis	1	Persisting UC and dilatation
Nephroureterectomy	3	Severe dysplasia

*UC *ureterocele*; VUR *vesicoureteral reflux.
